# Beneficial Effects of Mixing Kentucky Bluegrass With Red Fescue *via* Plant-Soil Interactions in Black Soil of Northeast China

**DOI:** 10.3389/fmicb.2020.556118

**Published:** 2020-10-28

**Authors:** Fuchun Xie, Gaoyun Zhang, Qianjiao Zheng, Kemeng Liu, Xiujie Yin, Xiaoyang Sun, Shah Saud, Zhenjie Shi, Runli Yuan, Wenjing Deng, Lu Zhang, Guowen Cui, Yajun Chen

**Affiliations:** ^1^ College of Horticulture and Landscape Architecture, Northeast Agricultural University, Harbin, China; ^2^ Beijing Oriental Garden Environment Co., Ltd, Beijing, China; ^3^ College of Animal Science and Technology, Northeast Agricultural University, Harbin, China

**Keywords:** turf mixture, turfgrass quality, plant-soil interaction, black soil, *Poa pratensis* L., rhizosphere microbial communities

## Abstract

Continuous monoculture of cool-season turfgrass causes soil degradation, and visual turf quality decline is a major concern in black soil regions of Northeast China. Turf mixtures can enhance turfgrass resistance to biotic and abiotic stresses and increase soil microbial diversity. Understanding mechanism by plant-soil interactions and changes of black soil microbial communities in turf mixture is beneficial to restoring the degradation of urbanized black soils and maintaining sustainable development of urban landscape ecology. In this study, based on the previous research of different sowing models, two schemes of turf monoculture and mixture were conducted in field plots during 2016–2018 in a black soil of Heilongjiang province of Northeast China. The mixture turf was established by mixing 50% Kentucky bluegrass “Midnight” (*Poa pratensis* L.) with 50% Red fescue “Frigg” (*Festuca rubra* L.); and the monoculture turf was established by sowing with pure Kentucky bluegrass. Turf performance, soil physiochemical properties, and microbial composition from rhizosphere were investigated. Soil microbial communities and abundance were analyzed by Illumina MiSeq sequencing and quantitative PCR methods. Results showed that turfgrass quality, turfgrass biomass, soil organic matter (SOM), urease, alkaline phosphatase, invertase, and catalase activities increased in PF mixture, but disease percentage and soil pH decreased. The microbial diversity was also significantly enhanced under turf mixture model. The microbial community compositions were significantly different between the two schemes. Turf mixtures obviously increased the abundances of *Beauveria*, *Lysobacter*, *Chryseolinea*, and *Gemmatimonas* spp., while remarkably reduced the abundances of *Myrothecium* and *Epicoccum* spp. Redundancy analysis showed that the compositions of bacteria and fungi were related to edaphic parameters, such as SOM, pH, and enzyme activities. Since the increasing of turf quality, biomass, and disease resistance were highly correlated with the changes of soil physiochemical parameters and microbial communities in turf mixture, which suggested that turf mixture with two species (i.e., Kentucky blue grass and Red fescue) changed soil microbial communities and enhanced visual turfgrass qualities through positive plant-soil interactions by soil biota.

## Introduction

Lawn is an indispensable part of urban greening, which can provide many types of benefits to human beings, environmentally, esthetically, recreationally, economically, sociologically, and psychologically/physiologically (Cameron et al., 2012; Monteiro, 2017), whose value to human wellbeing is an increasingly seen feature (Smith and Fellowes, 2014). Therefore, turfgrass is widely used in the world. For instance, Turf dominates in urban and suburban green spaces in Canada and the United States (Smith and Fellowes, 2014); turfgrass occupies about 50 million acres in the form of residential turf in the United States, such as athletic fields, golf courses, highway roadsides, cemeteries, and parks (Ghimire et al., 2019). Turf not only makes up a substantial fraction of landscape in urbanized areas of many parts of the world (Blancomontero et al., 1995; Gaston et al., 2005) but also has important ecological functions in synthesizing organic matter, maintaining biodiversity, and preventing soil erosion. However, current turfgrass sowing is often dominated by monoculture, which has some disadvantages in wear tolerance, coverage, resistance, and requiring more high-energy inputs with chemical fertilizers, pesticides, and water consumption (Helfand et al., 2006; Pooya et al., 2013). The monoculture for turf often reduces turf visual quality and causes potential soil degradation and environmental problems. While, mixed sowing models in which at least two plant species are mixed-sown in the same field at the same time are reported to enhance plant diversity at the field scale, maintain multiple ecosystem functions, such as the efficient use of water, soil, light, heat, and natural resources (Dunn et al., 2002; Pooya et al., 2013; Wahbi et al., 2016; Li et al., 2019), and control plant diseases (Zhu et al., 2000; Paulsen et al., 2006; Sapoukhina et al., 2010), therefore, adopting turfgrass mixture may be beneficial to solve some problems existing in turf monoculture.

Mixture cultivation has been testified to be positively associated with yield in agriculture (Trenbath, 1974; Agegnehu et al., 2006; Pan et al., 2008; Sturludottir, 2011), such as wheat mixture (Cai et al., 2019), millet and sorghum mix-cropping (Iijima et al., 2016), grass-alfalfa mixture (Berdahl et al., 2001), multiple-tree planting (Zemp et al., 2019), and multi-species grass mixture (Bouman et al., 2010; Acharya et al., 2012). Additionally, recent studies have showed effects of the mixture on plant growth by plant-soil interaction (Zhao et al., 2015; Granzow et al., 2017; Marron and Epron, 2019). Granzow (2017) reported that wheat-faba bean mixtures can influence plant growth by altering the soil fertility and increasing interactions among microbial communities, enzyme activities, and soil substrates. Likewise, Zhao (2015) found that grass-legume mixtures improved richness and diversity of microorganisms and also increased the content of soil nitrogen as well as plant biomass. Hence, the mixture is beneficial to ecological and economical services by improving plant growth quality, increasing soil nutrition, and changing microbial communities. However, little is specified about plant-soil interaction in the mixed turfgrass model.

Some studies reported that these biological processes of plant-soil interaction in mixed model were mainly driven by soil microbial activities (Granzow et al., 2017; Zhou et al., 2017; Nivelle et al., 2018). Microorganisms play a vital role in the decomposition and synthesis of soil organic matter (SOM), the release and fixation of nutrients, and various pollutants conversion in terrestrial ecosystems (Wang et al., 2017a; Frouz, 2018; Gabrijel et al., 2019). For example, Silvestro (2017) verified that zero tillage soil fungal culture is supported by multiple cropping regimes. Wahbi (2016) reported that wheat-faba bean mixture was beneficial to improving the efficiency of nitrogen utilization by the symbiotic relationship between legumes and nitrogen-fixing bacteria. Therefore, revealing changes of soil microorganisms in mixture model is helpful to formulate cultural practices to improve soil conditions and increase plant quality under the background of sustainable ecological development (Duchene et al., 2017; Zeng et al., 2019). However, to date, the effect of soil microorganisms in the mixed model mainly focuses on crops; the alternation of the microbial community composition and diversity in mixed turf soil have not been fully investigated, especially impact of the mixture turf on microbial activities in black soil regions in China.

Black soil (Mollisols) is one of the most important resources for vegetation and has crucial functions in protecting environment and landscape (Yao et al., 2017); black soil is mainly distributed in North and South America, Russia, Northeast China, and Ukraine (Liu et al., 2012, 2015; Yao et al., 2017). Some of the black soils have severely degraded during the past several decades in China due to growing population, urban sprawl, long-term poor management, and soil erosion (Hu et al., 2017). Turfgrass as an artificial vegetation is recognized to be beneficial to restoring the degraded soil and maintaining sustainable development of urban ecology (Monteiro, 2017). However, continuous monoculture of cool-season turfgrass causes soil degradation and visual turf quality decline in black soil regions of Northeast China. Turf mixtures can enhance resistance of turfgrass to biotic and abiotic stresses (Yin, 2017) and increase soil microbial diversity. Hence, revealing mechanism by the plant-soil interactions and changes of black soil microbial communities in turf mixtures is an urgent problem.

Kentucky bluegrass (*Poa pratensis* L.) as a vital cool-season turfgrass (Chen et al., 2019), which has advantages in cold resistance, dark green color, and strong tillering ability, is widely used in cold regions of the world for landscaping (Shah et al., 2014, 2016, 2017). However, Kentucky bluegrass grows slowly and is easily invaded by weeds; thus, it is usually mixed with other turfgrass species for turf establishment in practice. In particular, the mixed-sown model of Kentucky bluegrass-Red fescue is popular in turf (Dunn et al., 2002). Red fescue (*Festuca rubra* L.) is a perennial turfgrass, which has strong reproduction, cold resistance, and early rejuvenation (Chen et al., 2018). Our previous research found that comprehensive quality under turfgrass mixture with 50% Kentucky bluegrass and 50% Red fescue (*P. pratensis* L. and *F. rubra* L., PF) was the highest among four sowing models (Supplementary Table S1), with obviously improved visual quality, reduced disease, and increased biomass compared to Kentucky bluegrass (*P. pratensis* L., PP) monoculture (Yin, 2017). Nevertheless, it is necessary to deepen the understanding of microbial community differences between PF mixed sowing and PP monoculture in the black soil regions in China, in the context of dynamical interaction between aboveground and belowground organisms.

For this reason, we first investigated the turf growth characteristics in PF and PP models. In the black soil, physicochemical properties and enzyme activities were determined through individual experiment methods; diversities and compositions of soil microbial communities were analyzed using high-throughput sequencing; quantitative PCR was used to determine absolute abundances of bacteria and fungi. We hypothesized that PF mixed sowing is beneficial to restoring the degradation of urbanized black soils and increasing visual turf quality by positive plant-soil interactions. The hypothesis was verified through exploring three questions: (1) whether turfgrass mixture improves physicochemical properties and enzyme activities in the black soil regions, (2) whether the mixture increases abundance and diversity of black soil microbial community, and (3) which microbial communities associate with improving visual quality and reducing disease of turfgrass.

## Materials and Methods

### Site Description and Experimental Design

The experimental site was located at the Experimental Station of Northeast Agricultural University (45°41'N, 126°37'E), Harbin, Heilongjiang Province, China. The maximum and minimum temperatures are 34.5 and −38.3°C, respectively. Before the experiments started, the field was wasteland for more than 10 years. The soil is a typical black soil (Mollisol; Yao et al., 2017). The SOM, ammonium nitrogen (NH_4_^+^-N), available phosphorus (AP), and available potassium (AK) contents of the soil were tested to be 2.4%, 8.2, 57.6, and 87 mg kg^−1^, respectively, and the soil pH was 6.8.

The field experiment was carried out from April 2016 to October 2018, which was set mixture and monoculture. The mixture model (PF) was designed to sow a mixture of 50% Kentucky bluegrass “Midnight” (*P. pratensis* L.) and 50% Red fescue “Frigg” (*F. rubra* L.); and the monoculture model (PP) was sown pure Kentucky bluegrass. The cultivar “Midnight” was supported by Beijing Bright Grass Industry Company, and the “Frigg” was provided by Norwegian Institute for Bio-economy Research in Norway. Sowing was conducted in May 2016, and seeding rate was 25 g m^−2^. There were four replicates for each model, and per plot area was 2.25 m^2^. The randomized block design was used in all plots. The turfgrasses in experimental plots were managed in normal maintenance of water and fertilizer during 2016–2018.

### Aboveground Indices of Turfgrass

#### The Visual Turfgrass Quality

Turf quality was visually assessed once a week on all plots according to Chen et al. (2018). The rating scales used were 1 (very poor) to 9 (excellent), and the lowest acceptable value was 5 (Xie et al., 2020).

#### Percentage of Turfgrass Disease

The main pathogens in turfgrass plots were rust (*Puccinia* spp.), powdery mildew (*Erysiphe graminis*), and brown spot (*Rhizoctonia solani*). Therefore, we investigated the percent of these pathogens in PP monoculture and PF mixture turfgrasses from July to October 2018. Three quadrats (100 cm^2^) were randomly selected in each plot. Infected tillers in each quadrat were recorded every week. The percentage of disease in PP and PF was calculated as: (Infected tiller numbers/total tiller numbers) × 100%.

#### Turfgrass Density

Three quadrats (100 cm^2^) were randomly selected in each plot; tiller numbers in each quadrat were recorded in September 2018. Turfgrass density (tiller numbers/cm^2^) was calculated as: total tiller numbers/total quadrat areas.

#### Root Biomass

Three quadrats (5 cm^2^) in each plot were randomly selected for evaluating biomass underground from 0 to 20 cm surface depth in September 2018. Underground roots were then rinsed and dried at 65°C for 48 h; dry weight of samples were weighed and calculated in two sowing models. Root biomass (g/cm^2^) is calculated as: total root dry weight/total quadrat areas.

#### Soil Sampling

For each plot, five soil cores at random points from 0 to 15 cm depth were collected by a 2.5 cm diameter in September 2018. These five cores of each plot were then pooled together to form one combined sample, and four samples were obtained from per sowing model. The eight soil samples from mixture and monoculture field were placed in an individual sterile plastic bag, which was packed inside an ice box and immediately brought to laboratory. Each soil sample was sieved and thoroughly homogenized by a 2-mm sieve. For soil chemical property analysis, one part of these new sampled soils was air-dried (<30°C); one part was refrigerated at 4°C and used for soil enzyme analysis; and the other portion was stored at −80°C for DNA extraction.

#### Soil Physicochemical Properties and Enzyme Activities

Soil pH was estimated in soil suspensions with deionized-distilled water (1:2.5, w/v; Rayment and Higginson, 1992). SOM content was measured using the classical potassium dichromate oxidation method (Nelson and Sommers, 1982). Soil moisture content was measured using gravimetry (Yao et al., 2017). NH_4_^+^-N and AP were separated with 2.0 M KCl and 0.5 M NaHCO_3_, respectively (Olsen et al., 1954; Lu, 1999). Using continuous flow analysis system (SAN++ SKALAR, The Netherlands) for measuring the concentration of NH_4_^+^-N and AP. AK was extracted with 1.0 M NH_4_OAc (Mehlich, 1984) and quantified using inductively coupled plasma-atomic emission spectrometry (ICPS-7500, Shimadzu, Japan).

Four kinds of soil enzymes including urease, alkaline phosphatase, invertase, and catalase were measured following the methods described by Halstead (1964), Johnson and Temple (1964), Frankenberger and Johanson (1983), and Floch (2009), respectively.

#### DNA Extraction, PCR Amplification and Illumine MiSeq Sequencing

Total DNA was extracted from each rhizosphere of sampled soils (0.25 g) using E.Z.N.ATM Mag-Bind Soil DNA Kit (Omega, China). Genomic DNA concentration was measured using a Qubit 3.0 DNA detection kit (Transgen, China). V3–V4 regions of the bacterial 16S rRNA gene and the ITS1 regions of the fungal rRNA gene were amplified with primer sets of F341 (CCTACGGGNGGCWGCAG)/R805 (GACTACHVGGGTATCTAATCC; Fadrosh et al., 2014) and ITS1F (5'-CTTGGTCATTTAGAGGAAGTAA-3')/ITS2R (5'-GCTGCGTTCTTCATCGATGC-3'), respectively (White et al., 1990).

In brief, a 30 μl reaction consisting 15 μl of 2× Taq master Mix (Vazyme Biotech Co., Ltd), 1.0 μl of 10 μM of each primer, 12 μl of sterilized Milli-Q water, and 20 ng of isolated soil DNA was performed for each PCR reaction. The amplification conditions were initial denaturation at 95°C for 3 min, and then five cycles at 94°C for 30 s, 45°C for 20 s, and 65°C for 30 s, followed by 25 cycles at 94°C for 30 s, 55°C for 30 s, 72°C for 30 s, extended at 72°C for 5 min and 4°C for keeping. PCR items were then purified using MagicPure Size Collection DNA Beads (Thermo Fisher Science, United States), and a Qubit 3.0 DNA detector kit was used to measure distilled amplicons (TransGen Biotech, China), and was prepared properly to obtain the equal concentration (20 pmol) in the final mixture at Sangon Biotech Co. Ltd., Shanghai, China; the mixture was then paired-end sequenced from fungal and bacterial amplicons on an Illuminina Miseq platform. The raw data of bacteria and fungi have been deposited in the GenBank short-read archive with the numbers SRP280328 and SRP281151, respectively.

#### Quantification of Bacterial and Fungal Abundances

In an IQ5 real-time PCR system (Bio-Rad Lab, LA, United States), the fungal and bacterial abundances were estimated by quantitative polymerase chain reaction system. Primers of ITS1F (TCCGTAGGTGAACCTGCGG)/5.8 s (CGCTGCGTTCTTCATCG; Chen et al., 2015) and 338F (CCTACGGGAGGCAGCAG)/518R (ATTACCGCGGCTGCTGG; Muyzer et al., 1993) were used to amplify the fungal ITS regions of the rRNA gene and the partial 16S rRNA genes, respectively, for the fungal and bacterial organisms.

Standard curves were produced from a clone with a 10-fold serial dilution of the ITS or 16S rRNA genes. Melting curve analysis was used to testify the specificity of the products. Briefly, the 20 μl qPCR reaction consisted 10 μl of SybrGreen qPCR Master Mix (2×; High Rox), 0.4 μl of 10 μM each primer, 7.2 μl of sterilized MilliQ water, and 2.0 μl of standard DNA. The qPCR reaction was performed by following thermal conditions: 95°C for 30 s at initial steps, 45 cycles of 95°C for 15 s, 57°C for 20 s, and 72°C for 30 s. The initial copy number of the target gene was obtained using the cycle threshold (Ct) value of each sample to compare with the standard curves. Sterile Milli-Q water replace soil DNA in all reagents of negative controls. All samples were quantified in triplicate.

#### Statistical Analysis

Data of turfgrass growth, soil physicochemical characteristics, and total fungal and bacterial abundances in PF mixture and PP monoculture soils were compared using one-way ANOVA performed by SPSS ver. 21.0 software. Alpha diversity indices of fungi and bacteria among samples, including Chao1, operational taxonomic units (OTUs), Shannon, and inverse Simpson indices, were generated in QIIME. Fungal and bacterial alpha diversities, and the relative abundances of different taxa levels were compared between treatments by the Welch’s *t*-test performed by STAMP ver. 2.1.3 software. For beta diversity analysis, weighted UniFrac distances and Bray-Curtis distances were determined using QIIME and “vegan” package in “R” (R v.3.2.0), respectively.

To classify variations in the composition of the bacterial population, principal coordinates analysis (PCoA) was used; redundancy analysis (RDA) was conducted to establish important or significant environmental variables specific to the microbial community, which were performed in the R community using the “vegan” package (R v.3.2.0). Mantel test was conducted in “ade4” package “R” (version 2.1.3); Spearman correlations between environmental factors and relative abundances of the microbial community were determined in “psych” package “R” (version 3.3.1), respectively.

## Results

### Plant Growth Characteristics in Two Sowing Models

**Figure 1:**
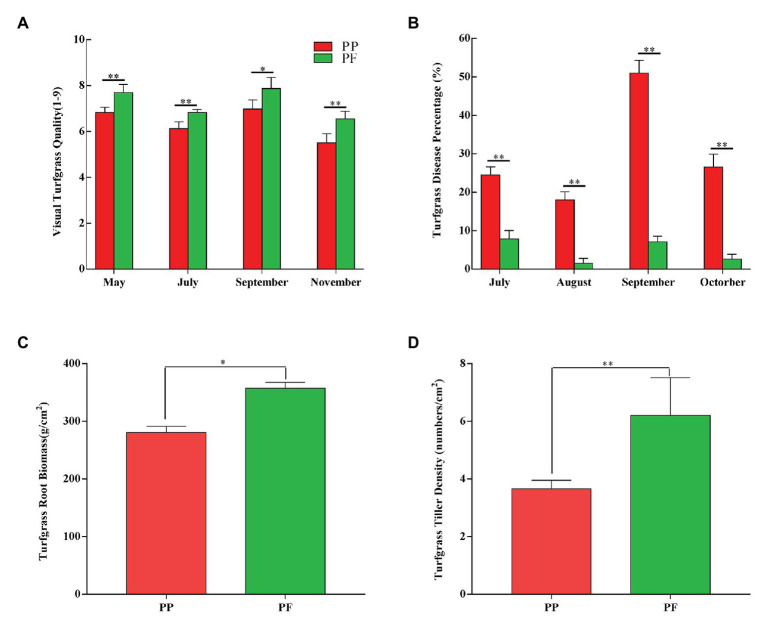
Effects on visual turfgrass quality **(A)**, disease percentage **(B)**, root biomass **(C)**, and tiller density **(D)** in PF mixture and PP monoculture. ^*^ and ^**^represent significant difference between PF mixture and PP monoculture (*p* < 0.05 and *p* < 0.01, one-way ANOVA, *n* = 4).

Visually assessed turfgrass quality in PF and PP was shown in Figure 1A. The mean values of turfgrass quality of PF and PP were 7.24 and 6.36 during the growing stage, respectively; and the visual turf quality of the PF was better than that of PP during different turfgrass periods (May–November; *p* < 0.05). Particularly, during the main period (May–July) of the assessment, the turfgrass quality between PF and PP was significantly different (*p* < 0.01).

The disease percentage of PF and PP was shown in Figure 1B and Supplementary Table S2. The disease percentage of PF was very low during the sensitive period of July–October. Compared with PP, the disease percentage of PF was significantly lower during the same period (*p* < 0.01). The highest and the lowest disease percentage of PF were 7.87 and 1.50%, respectively, while those of PP were 51.0 and 18.0%, which showed that the PF significantly improved the disease resistance of turfgrass.

The turfgrass root biomass of PF and PP was significantly different (*p* < 0.05; Figure 1C). The root biomass was 357.7 g cm^−2^ in the PF, which increased by 27.43% compared with the PP monoculture. In addition, the aboveground density of PF increased by 69.39% compared with that of PP, and the difference between two models was significant (*p* < 0.01). The aboveground density of turfgrass in PF and PP were 6.2 and 3.66 tillers cm^−2^ (Figure 1D), respectively.

### Soil Physicochemical Properties and Enzyme Activities

Compared with the PP monoculture (Table 1), soil pH decreased by 6.7% and was close to 7, which was significant lower (*p* < 0.01), while PF mixture significantly increased SOM content in the soil compared with the PP (*p* < 0.01). Soil moisture varied slightly between the PF and the PP. Among those available soil nutrients tested, soil AP and AK contents increased more in the PF than in the PP, whereas NH_4_^+^-N decreased. This analysis showed that mixed sowing was beneficial to soil pH, SOM, soil moisture, AP, and AK but less affected on NH_4_^+^-N.

**Table 1:** Effects of PF mixture and PP monoculture on soil physicochemical properties.

	pH	SOM (g kg^−1^)	Moisture (%)	NH_4_^+^-N (mg kg^−1^)	AP (mg kg^−1^)	AK (mg kg^−1^)
PF	7.0 ± 0.09	3.2 ± 0.20	16.5 ± 0.10	9.6 ± 0.07	83.4 ± 0.53	79.9 ± 1.91
PP	7.5 ± 0.06	3.0 ± 0.21	16.3 ± 0.17	9.8 ± 0.21	82.9 ± 0.16	79.1 ± 0.72
ANOVA	*p* < 0.01	*p* < 0.01	*p* > 0.05	*p* > 0.05	*p* > 0.05	*p* > 0.05
*p*	*F* = 102.04	*F* = 130.59	*F* = 4.67	*F* = 4.44	*F* = 4.67	*F* = 0.77

Values are means ± SE, PP, and PF (*n* = 4).

Effects of the two sowing models on soil enzyme activities are summarized in Table 2. All indicators were remarkably higher in the PF than in the PP (*p* < 0.05). Urease activity was the most remarkable (*p* < 0.01) among the four activities, with an increase of 14.72%, while catalase activity was slightly higher (*p* < 0.01) in the PF than in the PP. Alkaline phosphatase activity in the PF and the PP was 52.25 and 49.88 mg g^−1^ 24 h^−1^, respectively. Invertase activity in the PF soil increased 14.30% than that of PP. In general, the PF was beneficial to improve soil enzyme activities.

**Table 2:** Effects of PF mixture and PP monoculture on soil enzyme activities.

	Urease (mg g^−1^ 24 h^−1^)	Catalase(ml g^−1^ 20 min^−1^)	Alkaline phosphatase(mg g^−1^ 24 h^−1^)	Invertase(mg g^−1^ 24 h^−1^)
PF	24.93 ± 0.46	23.20 ± 0.47	52.25 ± 0.65	11.75 ± 0.31
PP	21.73 ± 0.66	22.03 ± 0.73	49.88 ± 0.85	10.28 ± 0.53
ANOVA	*p* < 0.01	*p* < 0.05	*p* < 0.01	*p* < 0.01
*p*	*F* = 63.50	*F* = 7.31	*F* = 19.69	*F* = 23.36

Values are means ± SE, PP, and PF (*n* = 4).

### Soil Fungal and Bacterial Total Abundance

**Figure 2:**
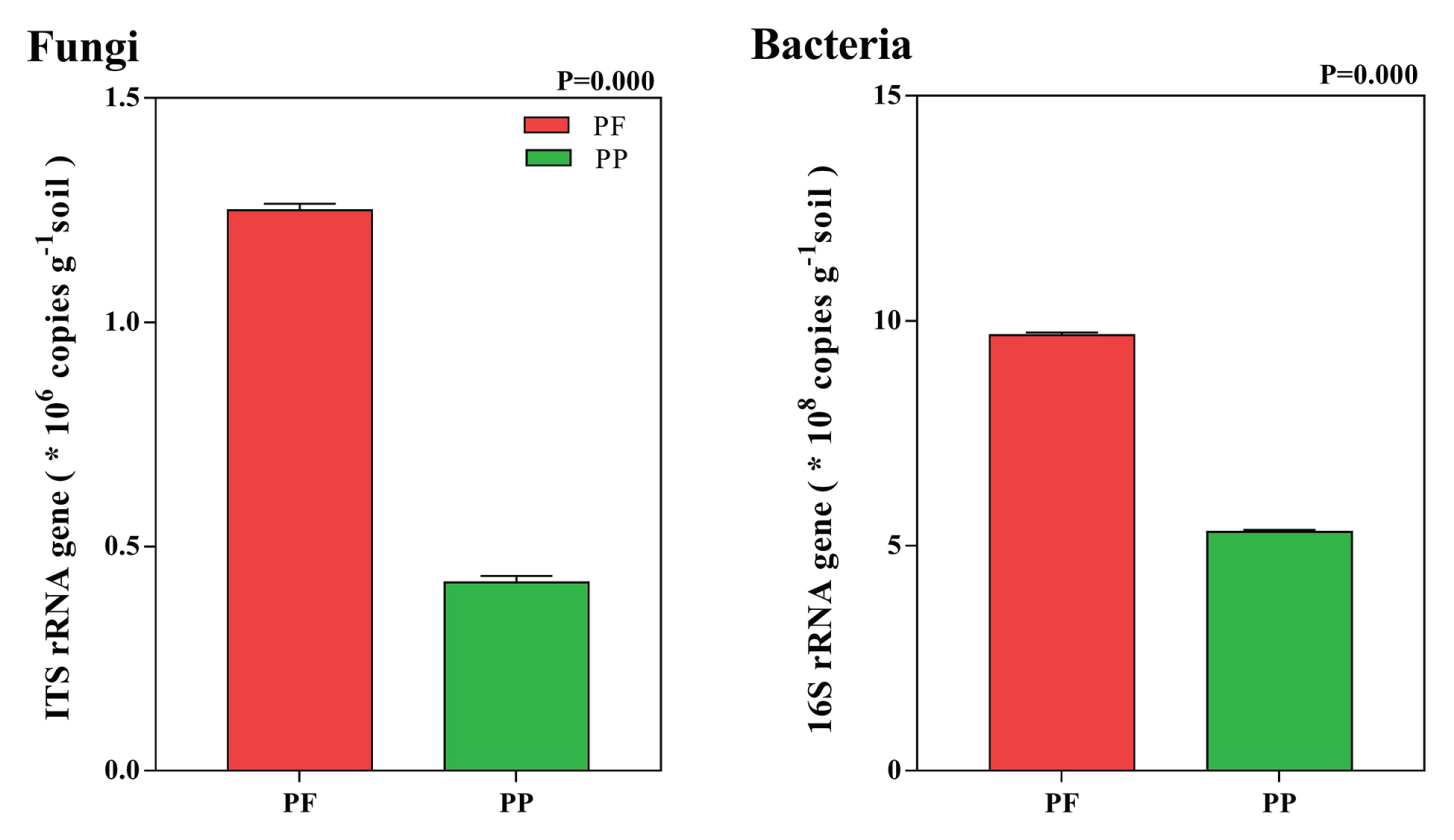
Total abundance of fungil and bacteria in PF mixture and PP monoculture soils.

The abundance of fungi in the PF and the PP was 1.26 × 10^6^ and 0.42 × 10^6^ copies g^−1^ soil, respectively, while the bacterial abundance was 9.69 × 10^8^ and 5.32 × 10^8^ copies g^−1^ soil, respectively. Compared with PP monoculture, PF mixture increased total fungal and bacterial abundances in the soil (*p* < 0.05). Total fungi and bacteria abundances in the PF soil were 2.98 and 1.82 times as many as in the PP soil, respectively (Figure 2).

### Taxonomic Classification and Relative Abundance of Fungi and Bacteria

Illumina Miseq sequencing yield 410,622 quality fungal sequences and 449,777 quality bacterial sequences across all soil samples, with 44,512–71,946 fungal sequences (mean = 51,327) and 45,502–71,303 bacterial sequences (mean = 56,222) per sample, and 2,757 fungal and 14,224 bacterial OTUs were obtained from fungal and bacterial sequences. For ITS1 regions and the 16S rRNA genes, average read lengths were 238 and 415 bp, respectively (Supplementary Table S3). For fungal and bacterial communities, Good’s coverage in each sample, which represents the captured diversity, was 99.34 ± 0.08 and 94.26 ± 0.17%, respectively (Supplementary Figure S1). Rarefactions curves of OTUs with 97% of all samples sequence similarities approached to the saturation plateau (Supplementary Figure S2), ANOSIM statistic R was close to 1 (*p* < 0.05), indicating that between groups difference was significantly higher than within-groups difference (Supplementary Figure S3).

### Taxonomic Characteristics of Fungal Communities

**Figure 3:**
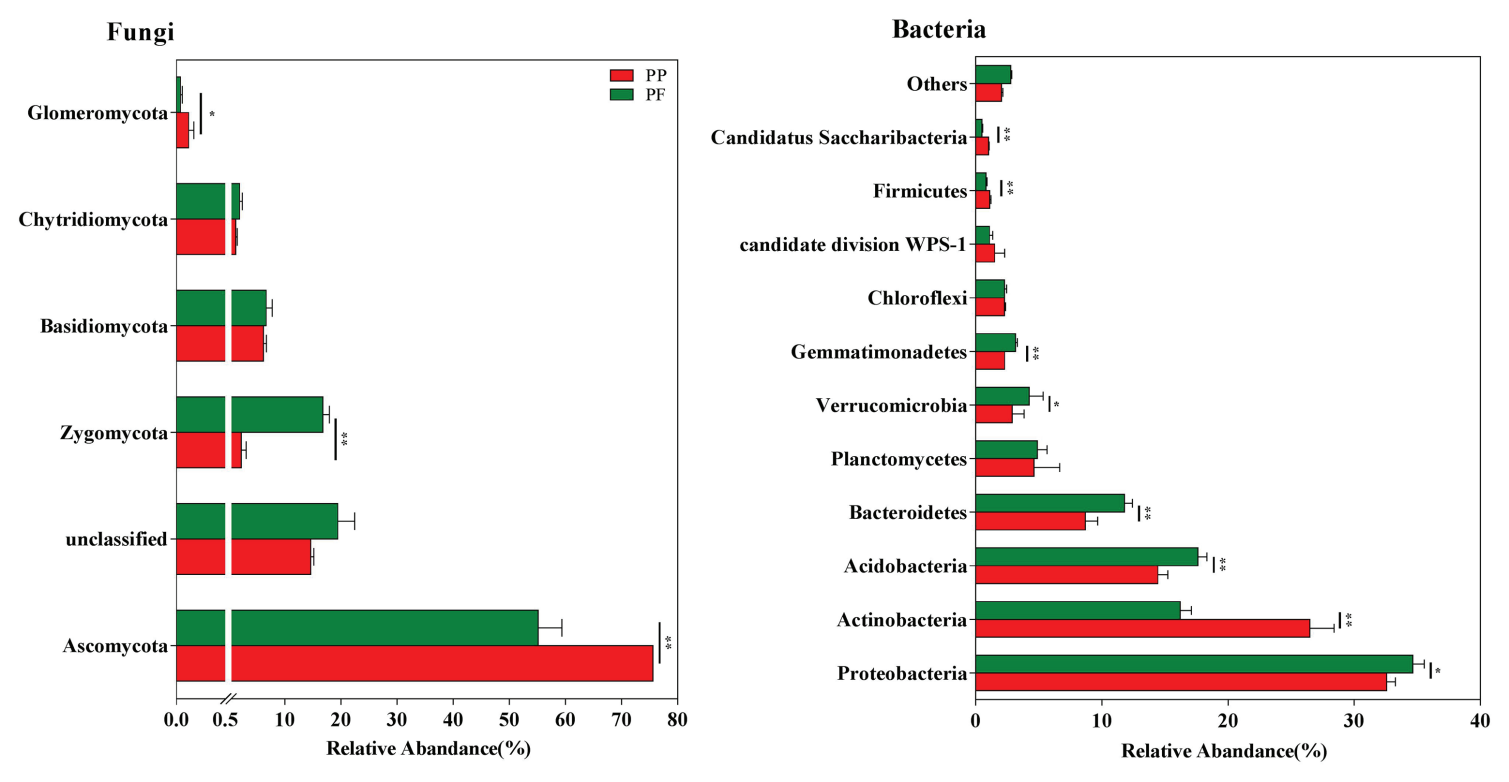
The effects of PF mixture and PP monoculture on relative abundances of fungal and bacterial phyla in rhizosphere soil. ^*^ and ^**^represent significant difference between PF mixture and PP monoculture (*p* < 0.05 and *p* < 0.01, Welch’s *t*-test, *n* = 4).

Five fungal phyla were found, and 17.01% fungal sequences were unclassified at the phyla level across all samples (Figure 3). Ascomycota (75.51 and 59.28% of sequences for the PP and the PF soils, respectively) and Zygomycota (2.26 and 16.81% of sequences for the PP and the PF soils, respectively) dominated fungal communities. Zygomycota were much more numerous in the PF soil than that of the PP soil (*p* < 0.01), whereas Ascomycota and Glomeromycota showed an opposite trend (*p* < 0.05; Figure 3).

**Figure 4:**
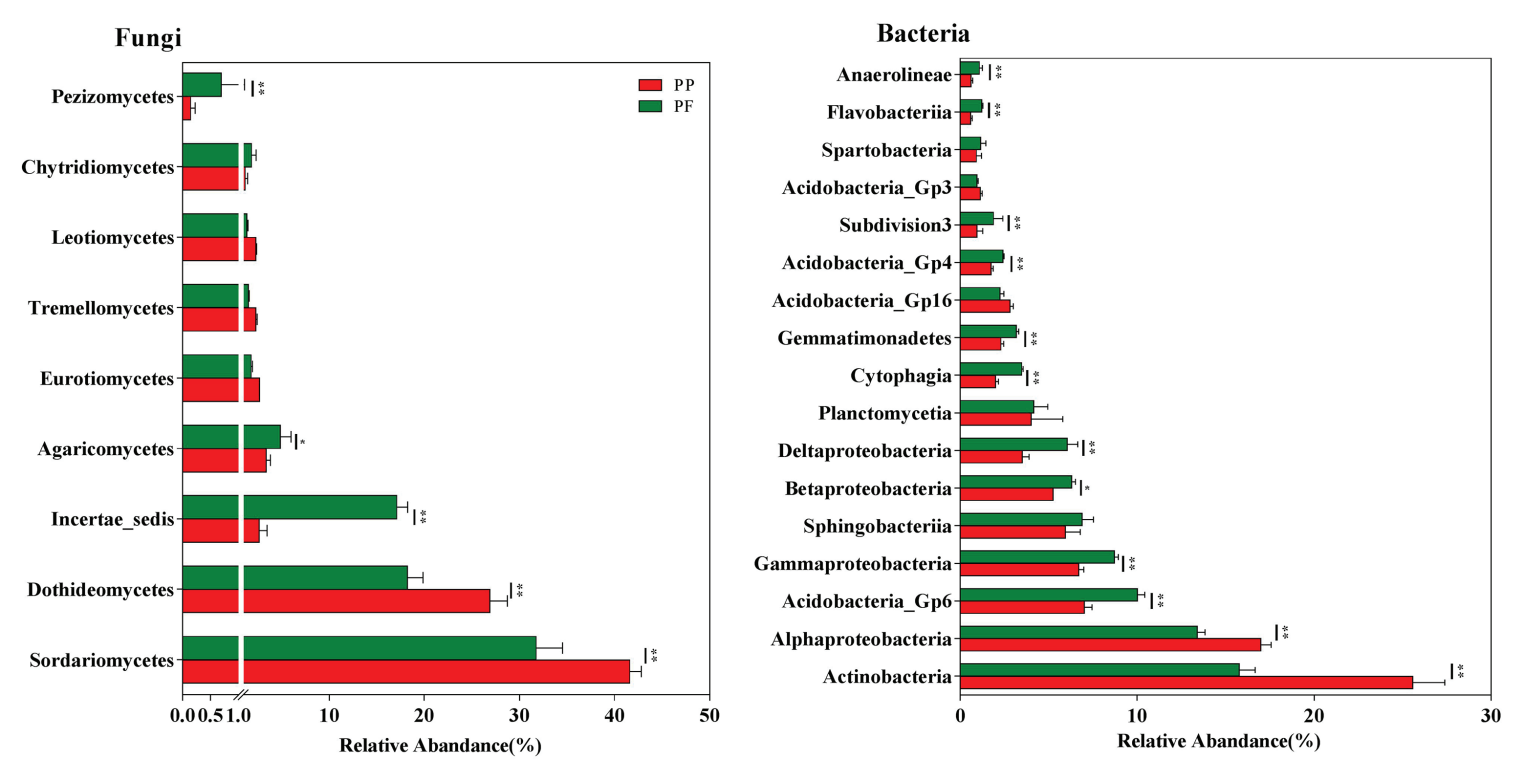
The effects of PF mixture and PP monoculture on relative abundances of main fungal and bacterial classes in rhizosphere soil. Fungal and bacterial classes with relative abundances > 1% are shown in at least one treatment. ^*^ and ^**^represent significant difference between PF mixture and PP monoculture (*p* < 0.05 and *p* < 0.01, Welch’s *t*-test, *n* = 4).

Sixteen fungi taxa were detected at the class level. Across all samples, Sordariomycetes, Dothideomycetes, Incertae_sedis, Agaricomycetes, Eurotiomycetes, Tremellomycetes, Leotiomycetes, Chytridiomycetesm, and Pezizomycetes were dominant fungal classes (relative abundance > 1.0%; Figure 4). Relative abundances of the fungal class Incertae_sedis, Agaricomycetes, and Pezizomycetes were higher, while Sordariomycetes and Dothideomycetes obviously reduced in the PF soil (Figure 4).

**Figure 5:**
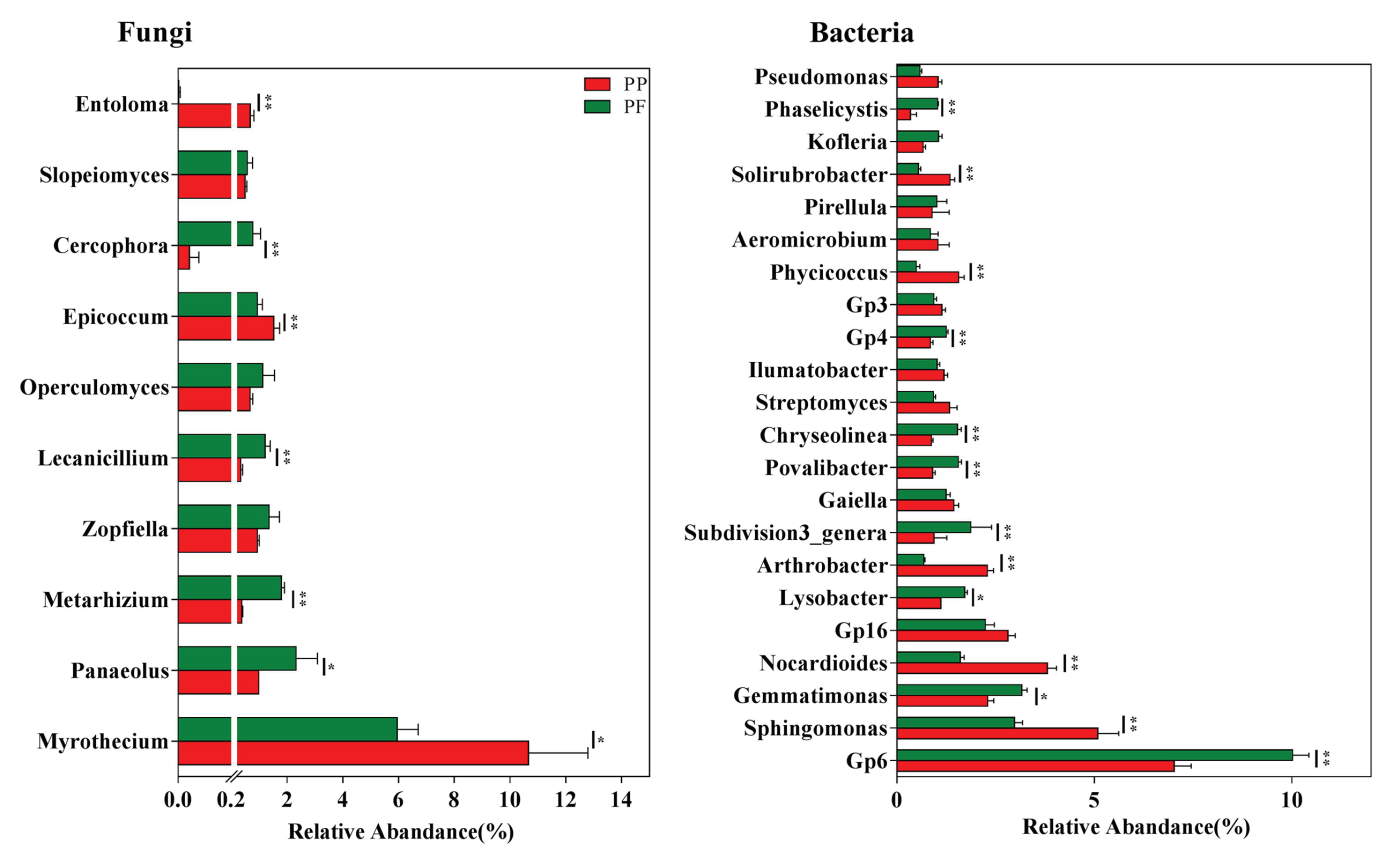
The effects of PF mixture and PP monoculture on relative abundances of mian fungal and bacterial genera in rhizosphere soil. Fungal genera (relative abundances > 0.5%) and bacterial genera (relative abundances > 1%) are shown in at least one treatment. ^*^ and ^**^represent significant difference between PF mixture and PP monoculture (*p* < 0.05 and *p* < 0.01, Welch’s *t*-test, *n* = 4).

The detection of more than 160 fungal genera in this study was revealed by further taxonomic classification at the genus level (data not shown). Of these, 29 genera with the relative abundance > 0.1% were observed in all soil samples, and the relative abundances of these genera in the PF soil were obviously different from that in the PP soil (Figure 5 and Supplementary Figure S4). We further analyzed the association of fungal genera (relative abundances > 0.1%) with the PF and the PP soils. *Metarhizium*, *Zopfiella*, *Lecanicillium*, *Operculomyces*, *Cercophora*, *Cryptococcus*, and *Beauveria* spp. were more abundant in the PF soil, whereas *Myrothecium*, *Epicoccum*, *Trichoderma*, and *Waitea* spp. were more dominant in the PP soil (Figure 5 and Supplementary Figure S4). Additionally, *Heteroconium* and *Mortierella* were unique in the PF soil (Supplementary Figure S4).

### Taxonomic Characteristics of Bacterial Communities

A total of 23 different phyla were classified from bacterial sequences in all samples, and 1.35% of bacterial sequences were unclassified (Figure 3). The phyla Proteobacteria (33.61%), Actinobacteria (21.36%), Acidobacteria (16.02%), and Bacteroidetes (10.24%) were remarkably associated with bacterial sequences. At relatively high abundances with 1.0% < relative abundance < 10.0%, Planctomycetes, Verrucomicrobia, Gemmatimonadetes, Chloroflexi and Firmicutes were observed. However, the changes in the relative abundances of these groups were different: Proteobacteria, Acidobacteria, Bacteroidetes, Verrucomicrobia, and Gemmatimonadetes were evidently more in the PF soil, while Actinobacteria and Firmicutes were more in the PP soil (*p* < 0.05).

Taxonomical classification obtained more than 60 bacterial classes in all samples (Figure 4). Among them, Actinobacteria, Acidobacteria_Gp6, Alphaproteobacteria, Gammaproteobacteria, Sphingobacteriia, Betaproteobacteria, and Deltaproteobacteria (average relative abundance > 5.0%) were prominent (Figure 4). Planctomycetia, Cytophagia, Gemmatimonadetes, Acidobacteria_Gp16, Acidobacteria_Gp4, Subdivision3, Acidobacteria_Gp3, Spartobacteria, Flavobacteriia, and Anaerolineae were also found at relatively high abundances (1.0% < relative abundance < 5.0%). The relative abundance of bacteria class Acidobacteria_Gp6, Gammaproteobacteria, Betaproteobacteria, Deltaproteobacteria, Cytophagia, Gemmatimonadetes, Acidobacteria_Gp4, Subdivision3, Flavobacteriia, and Anaerolineae increased while those of Actinobacteria and Alphaproteobacteria reduced in the PF soil (*p* < 0.05).

The existence of more than 570 bacterial taxa in the PF and the PP soils were revealed by taxonomic classifications at the genus level (data not shown). Of these, 22 genera with relative abundance > 1.0% were observed among all soil samples, accounting for more than 39.9% of the total bacterial sequences. The PF soil had higher relative abundances of *Gp6*, *Gemmatimonas*, *Lysobacte*, *Povalibacter*, *Chryseolinea* spp. (*p* < 0.05; Figure 5), or *Ohtaekwangia*, *Flavobacterium*, *Reyranella*, *Chitinophaga*, *Phaselicystis*, *Pedobacter*, and *Sulfuritalea* (0.5% < relative abundance < 1.0%) compared with PP soil (Supplementary Figure S4). However, the relative abundances of *Sphingomonas* and *Nocardioides* spp. decreased in the PF soil.

### Alpha and Beta Diversities of Bacterial and Fungal Communities

**Figure 6:**
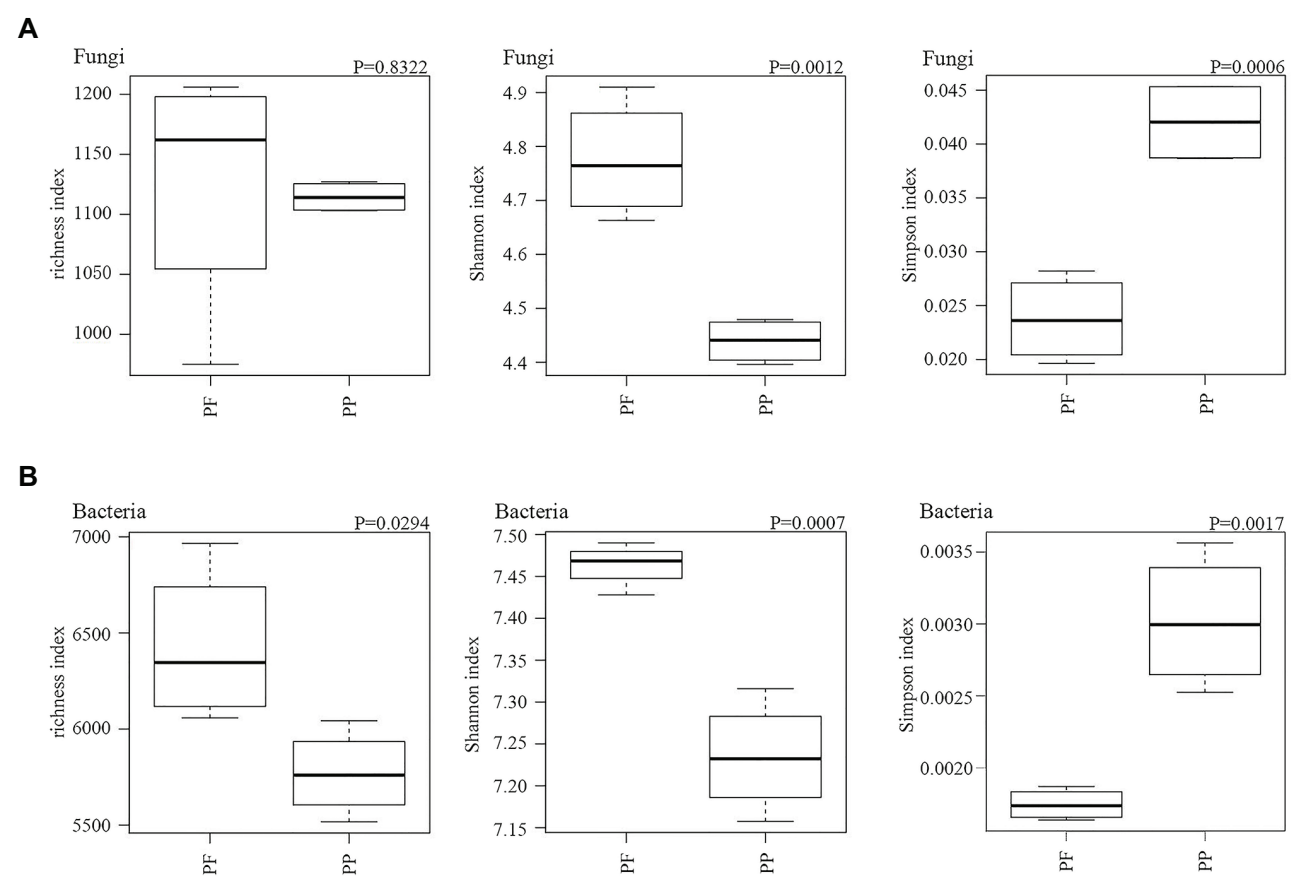
The effects of PF mixture and PP monoculture on diversity and richness indices of soil fungal **(A)** and bacterial **(B)** communities in rhizosphere soil. *p* < 0.05 (Welch’s *t*-test, *n* = 4) represents significance between PF mixture and PP monoculture soils.

Fungal and bacterial richness, Simpson and Shannon’s indexes are summarized in Figure 6. The change of fungal richness in the PF and the PP soils was not obviously different; the Shannon index and inverse Simpson index were obviously higher in the PF soil than in the PP soil (*p* < 0.05). For the bacterial community the richness and the Shannon and inverse Simpson indices were also significantly increased in the PF soils (*p* < 0.05).

**Figure 7:**
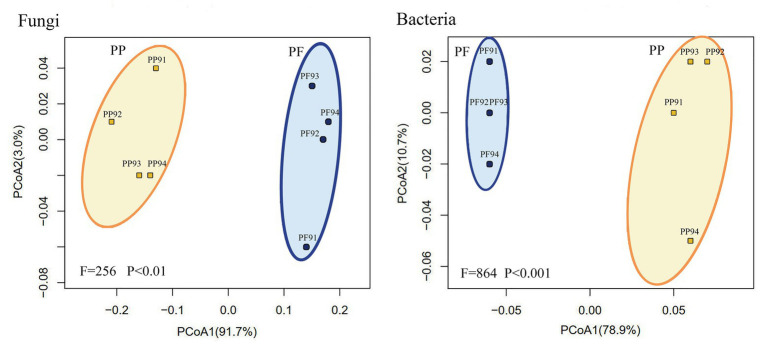
Change of fungal and bacterial community structures in PF mixture and PP monoculture soils from principal coordinate analysis.

As shown in Supplementary Figure S5 and Figure 7, the Bray tree plot and UniFrac PCoA revealed that community compositions of both fungi and bacteria in the PF soil were distinctly different from those in the PP soil, which demonstrated that mixed sowing of the PF evidently changed soil microbial community structure.

### Relationships Between Soil Microbial Community Structure and Soil Physicochemical Properties

**Figure 8:**
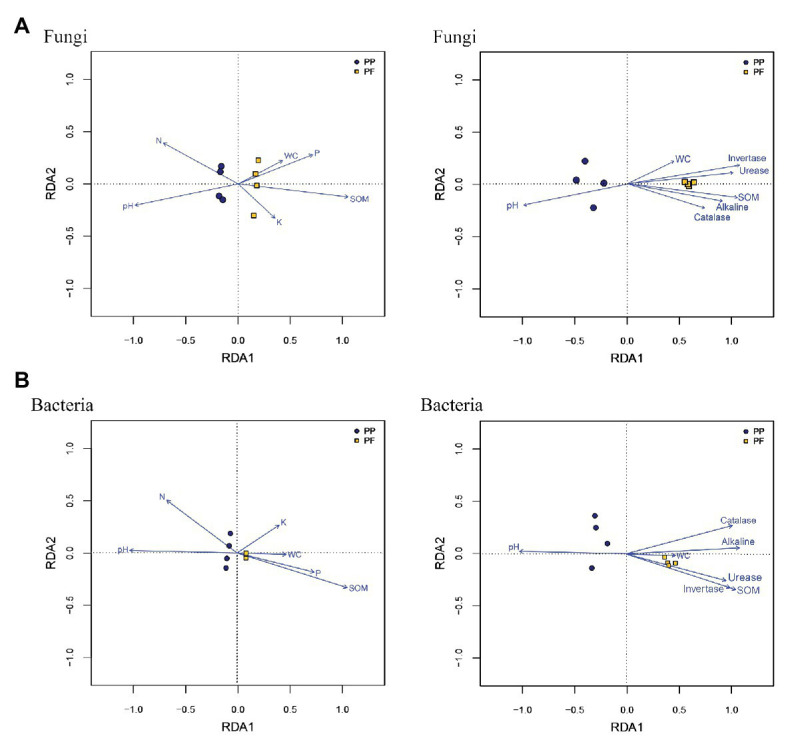
Relationship between community structures of soil fungi **(A)** and bacteria **(B)** and environmental factors from the redundancy analysis.

Mantel test and RDA analysis were performed to distinguish key drivers of soil microbial community composition and structure. The Mantel tests revealed that the composition of soil fungal communities was strongly associated with pH (*r* = 0.826, *p* = 0.012), SOM (*r* = 0.909, *p* = 0.007), invertase (*r* = 0.973, *p* = 0.001), alkaline (*r* = 0.702, *p* = 0.024), and urease (*r* = 0.849, *p* = 0.010), and soil bacteria community structure was significantly correlated to pH (*r* = 0.776, *p* = 0.027), SOM (*r* = 0.952, *p* = 0.009), invertase (*r* = 0.875, *p* = 0.022), alkaline (*r* = 0.942, *p* = 0.007), and urease (*r* = 0.778, *p* = 0.031). As shown in RDA analysis, SOM, soil pH, and NH_4_^+^-N had longer arrows than soil moisture, AP, and AK, meanwhile four enzymes also had longer arrows compared soil moisture (Figure 8). Spearman’s rank correlation test found that abundances in soil fungal communities were significantly associated with pH (*r* = 0.759, *p* = 0.028), SOM (*r* = −0.952, *p* = 0.001), and NH_4_^+^-N (*r* = 0.880, *p* = 0.007), while soil bacterial community abundances were significantly related to SOM (*r* = 0.880, *p* = 0.007). In comparison, pH was positively related to the relative abundances of bacterial phylum Actinobacteria (*r* = 0.91, *p* = 0.0018), while pH was inversely related to the relative abundances of bacterial phyla Acidobacteria and Proteobacteria (*r* = −0.776, *p* = 0.023; and *r* = −0.725, *p* = 0.041, respectively). In genus, *Gp6* and *Gp4* were significantly associated with SOM (*r* = 0.950, *p* = 0.00028; and *r* = 0.941, *p* = 0.00049, respectively).

## Discussion

### Effects of PF Mixed Turfgrass on Biomass, Visual Quality, and Disease Resistance

Compared with the monoculture model, the mixture model increased turf biomass and visual quality in the black soil of northeast China, which was consistent with results in previous research (Simmons et al., 2011; Pooya et al., 2013; Granzow et al., 2017). Studies indicated that the mixture planted with two or more plant species improved soil soluble nutrient levels by the interaction between rhizosphere microorganisms and plants and subsequently increased biomass, and quality of plants (Eisenhauer, 2016; Li et al., 2019; Zeng et al., 2019). This kind of beneficial effect has been demonstrated in natural grasslands (Cardinale et al., 2012). Additionally, the mixture model reduced diseases infection rate, due likely to the lower abundances of soil pathogens and more abundances of antagonistic microorganisms in the mixture (Maron et al., 2011; Schnitzer et al., 2011).

### Responses of Soil Properties and Enzyme Activities to PF Mixture

Physical and chemical soil properties, which are generally considered as indicators of soil quality, are characterized as the capacity to maintain plant growth (Gong et al., 2019). The activities of soil enzyme are positively correlated with soil nutrient dynamics of soil nutrients and are indicators for assessing soil fertility conditions (Wang et al., 2015). In this study, SOM significantly increased in PF mixture soil compared to PP treatment. Additionally, the activities of urease, catalase, alkaline phosphatase, and invertase were also remarkably increased in PF mixture soil, which was consistent with previous studies that intercropping or mixed cropping significantly increased soil nutrient retention and enzyme activities (Xia et al., 2013; Yang et al., 2013; Gong et al., 2019). SOM is usually regarded as one of the key indicators of soil nutrient and health, while debris and root biomass normally contribute to the soil fertility (Sims et al., 1978; Wang et al., 2016, 2020; Ondrasek et al., 2019). More tillers and root biomass in PF mixture may be beneficial to the accumulation of SOM. Additionally, soil enzymes catalyze the transformation of complex organic matter to simple inorganic compounds in soil and participate soil biochemical processes (Lei et al., 2010). Baligar et al. (2005) and Wang (2015) manifested that the mixture system increased urease activity in soil because of higher microbial abundance and more humic substances in mixture soil compared to those in monoculture soil; catalase participated in the process of detoxification and had important functions in cellular antioxidant mechanisms (Gao et al., 2008; Huang et al., 2010); alkaline phosphatase is strongly associated with SOM and AP (Li et al., 2018); and invertase activity was beneficial to soil aggregate stability and cation exchange capacity as well as could increase contents of SOM and humus, in soil (Frankenberger and Johanson, 1983). In this study, the activities of four enzymes in PF mixture soil were significantly associated with SOM (Supplementary Table S4). Thus, SOM may contribute to the synthesis of these enzymes, which further showed that PF mixture had a great effect on soil enzyme activity. Moreover, SOM decomposition by microorganisms was the main way of soil nutrient turnover, resulting in affecting the biochemical characteristics of soil enzymes and regulating the secretion of enzymes (Tang et al., 2010; Peng et al., 2015). The difference of soil enzyme activity reflected that energy distribution and nutrient acquisition of microbial community were different *in situ* conditions (Gong et al., 2019), Therefore, PF mixture may enhance the nutrient supply, soil enzyme activities, and abundances of fungi and bacteria in the rhizosphere of the black soil, thereby the turfgrass quality and resisting ability to adverse environment were improved under PF mixture.

### Effects of PF Mixed Turfgrass on Soil Microbial Abundance, Diversity, and Structure

It has been an observation point that changes of microbial abundance, diversity, and structure in the soil occur due to diverse cropping systems (Latz et al., 2015; Zhou et al., 2018, Gong et al., 2019). This study examined some aspects of influences of the PF mixture model on soil microbial abundances and communities by qPCR and high-throughput sequencing. qPCR analysis showed that the PF mixture model had higher bacterial and fungal absolute abundances than the PP monoculture model (Figure 2). Alpha and beta diversity analyses showed that soil microbial diversity also significantly differed between the PP soil and the PF soil, with the PF mixture having higher bacterial and fungal diversities than the PP monoculture. This result is consistent with the general findings that the mixture has positive function in soil microbial community compositions (Zeng et al., 2019). As already shown in other studies, effects of mixed cropping or intercropping on microbial abundance, diversity, and community structure are remarkably different from those of monoculture soil (Zhou et al., 2017, 2018). Similarly, Latz (2015) reported that the mixture increases microbial community, diversity, and structure in the rhizosphere soil. Plant diversity can enhance black soil litter (carbon resource) and habitat heterogeneity, which would cascade upward to improve black soil aggregation and enhance contents of organic carbon and nitrogen, thereby changing the abundance and structure of soil fungi and bacteria (Eisenhauer et al., 2010; Eisenhauer, 2016). Another possible reason is that both the quantity and quality in root exudation between the PF and the PP led to the variations of fungal and bacterial communities (Li et al., 2016a,b). Therefore, the PF was more beneficial to microbial diversity, abundance, and structure than the PP (Stefanowicz et al., 2012). It is widely accepted that soil pH is the dominant factor for alterations of soil microbial abundance and structure (Berg and Smalla, 2009; Yao et al., 2011, 2017). Several studies indicated that edaphic physical parameters, especially soil pH, are key factors for changes of soil microbial communities and structures (Liu et al., 2014; Yao et al., 2017; Zhou et al., 2017). In this study, PF mixture model slightly decreased soil pH, which was closer to neutral. RDA analysis and Mantel test indicated that soil pH had significant correlation with soil microbial communities, which agreed on results observed by Zhou (2017), who stated that soil pH is associated with microbial communities in intercropping system. A similar finding was also reported by Zeng (2019). Therefore, soil pH, as modulated by the mixture model (Table 1), might be one of the factors for mediating bacterial and fungal abundances, diversity, and structure in the mixed sowing soil. To sum up, our results further indicated that mixed turf can improve bacterial and fungal abundances in the short term (Zeng et al., 2019), which supports our first hypothesis.

### Changes of Microbial Taxa in Response to PF Mixed Turfgrass

Microbial taxonomic composition slightly varied between PF and PP, whereas their relative abundances were prominently changed in this study. The change trends of bacterial and fungal community in the PF and the PP soils at the genera level were similar to those variations at the phyla level and the class level (Figures 3, 5). In addition to soil bacterial and fungal communities with the low relative abundance and the high variations at the general level, only the changes of relative abundance and composition at the phylum level were taken into deep discussions in this study. Actinobacteria, Acidobacteria, and Proteobacteria were mainly dominant bacterial phylum in the PF and the PP soils, which are consistent with previous studies of these three phyla being the top three prevalent bacterial phyla in a mixed cropping system (Zeng et al., 2017; Wang et al., 2019). Compared with PP, the PF mixture increased the relative abundance of Proteobacteria and Acidobacteria by 6.36 and 21.90%, respectively, whereas that of Actinobacteria decreased by 38.78% (Figure 3). The difference of relative abundance in Actinobacteria between PF mixture and PP monoculture might be much more related to the different quality of SOM released by turfgrass roots in PP and PF soils, resulting in changes of the groups with the ability to degrading specific organic substrates (i.e., lignocellulose degradation) as reported by several authors (Větrovský et al., 2014; Zhou et al., 2017, 2018). Moreover, Actinobacteria is positively related with the pH (Zhou et al., 2017); the pH value decreased by 6.67% from the PP to the PF, which might be one of the reasons for decreasing the relative abundance of Actinobacteria. Protecbacteria are copiotrophs that mainly consume labile organic substances and have high nutritional requirements (Aislabie et al., 2013), the higher SOM availability may be a major cause of the change in overall abundance of Proteobacteria in PF mixture model. The increase of abundance of Acidobacteria in the PF soil with high SOM content is contrary to previous findings that Acidobacteria prefer nutrient-poor soil condition (Fierer et al., 2007; Zhang et al., 2016; Zhou et al., 2017). The inconsistent results may be due to different subdivisions in Acidobacteria, which have different requirements for soil nutrients (Kielak et al., 2016). For example, members of the subdivisions 4 and 6 had high abundance in the fertile soil (Navarrete et al., 2015; Kielak et al., 2016). Another reason may be that other degrading bacteria are unable to survive in the black soil areas, there in which the lowest temperature was −32°C, or that Acidobacteria have the potential functions in the degradation of turfgrass residue and cellulose in rhizosphere soil (Pankratov et al., 2011; Zeng et al., 2019), suggesting that there are more plant residue and cellulose in the PF than those in the PP. Additionally, Acidobacteria are generally recognized to be sensitive to pH change and negatively related to pH values (Jones et al., 2009; Yao et al., 2017), the lower pH in PF mixture soil may contribute to the higher abundance of Acidobacteria.

Ascomycota and Zygomycota varied obviously and were more predominant fungal phyla in the PF and the PP. PF mixture decreased the relative abundance of Ascomycota in the rhizosphere soil by 20.82% but that of Zygomycota increased by 13.28% compared with the PP monoculture (Figure 3). This finding was consistent with previous researches that the relative abundance of soil fungal phyla changes due to their sensitivity to cropping models (Granzow et al., 2017; Wang et al., 2019). Kjoller and Struwe (1982) pointed that 78–90% of the total amount of decomposer organisms are fungi. Purahong et al. (2016) reported that saprophytic fungus mainly broke down litter into mineral nutrients that plants can absorb. Zygomycota may play a vital role in degrading turfgrass debris, thus the abundance of Zygomycota might be increased with more aboveground and underground biomass by PF mixture. Previous studies demonstrated that Ascomycota are key drivers for organic residue degradation in soils (Richardson, 2009; Ma et al., 2013). However, PF mixture increased content of SOM, while the relative abundance of Ascomycota decreased; the possible explanation might be that PF mixture decreased some potential plant pathogenic fungi which belong to Ascomycota phylum (Wang et al., 2019). For example, Sordariomycetes and Dothideomycetes, two classes of the Ascomycota (Figure 4), include some potential plant pathogenic fungi (Li et al., 2014; Gong et al., 2019), such as *Fusarium*. Interestingly, the relative abundance of *Fusarium* genus decreased by 46.67%, in a low frequency (<0.1%, data not shown), in the PF mixture. Thus, the lower abundance of Ascomycota may reduce the risk of diseases under PF mixture turfgrass.

Soil microorganisms can be classified as beneficial, neutral, and harmful for plant growth (Lynch, 1990; Zhou et al., 2018). The decrease of plant pathogens and increase of abundance of beneficial fungi and bacteria in soil contributed to positive interactions between plant and microorganisms (Latz et al., 2012; Zhou et al., 2017). The study showed that the PF mixture increased the relative abundances of *Metarhizium* (Moorhouse et al., 1992), *Lecanicillium* (Cabanillas and Jones, 2009), *Cryptococcus* (Köhl et al., 2015), *Beauveria* (Cabrera-Mora et al., 2019), *Chryseolinea* (Jatoi et al., 2019), and *Lysobacter* spp. (Chohnan et al., 2002; Qian et al., 2009), which have pathogen-antagonistic potential or the ability to metabolize specific products and enzymes to inhibit the survival and reproduction of pathogenic microorganisms in the soil of mixed turf, while the lower abundances of *Myrothecium* (Zhang et al., 2017) and *Epicoccum* (Stokholm et al., 2016) may have resulted in decreasing disease incidences in the PF mixture. Additionally, PF mixture obviously increased the relative abundance of *Gemmatimonas* in the rhizosphere soil compared with the PP monoculture. Members of *Gemmatimonas* are generally acknowledged to decompose cellulose and lignin as well as contribute to soil organic carbon accumulation (Bastian et al., 2009; Wang et al., 2017b; Xu et al., 2019). Additionally, the relative abundance of *Lysobacter* was also enriched in the PF soil. Postma (2011) and Chavez-Romero (2016) indicated that the members of *Lysobacter* spp. were more in rhizosphere soil with the higher clay content and lower C/N. Moreover, the abundance of saprophytic bacteria (*Povalibacter* spp. and *Chryseolinea* spp.) significantly increased in the PF soil, due likely to the abundance of its organic materials. Thus, the enhanced positive effects of black soil biota on turfgrass quality in the PF mixture may attribute to the decrease of grass pathogens and the increase of beneficial microorganisms.

## Conclusion

This study provided empirical evidence that plants mixture benefits plant growth through inducing positive plant-microbial interactions. PF increased the diversity of black soil bacterial and fungal communities. The increased positive effect of soil microorganisms on turfgrass quality in the mixed model was associated with the enhanced abundance of beneficial microorganisms and the decreased abundance of tufgrass pathogens. Changes in black soil physicochemical properties, such as increased content of SOM, AP, AK, NH_4_^+^-N, and enzyme activities in the PF, were correlated with changes in microbial community abundance and structure in the black soil between PF mixture and PP monoculture. Overall, the mixed turfgrass plays positive role in improving the black soil eco-quality and ecosystem services in northeast of China. Further studies are, however, needed to validate those findings, in light of broad-acre turf management practice for designing a sustainable and environment-friendly landscape. Moreover, our future research should pinpoint those specific microbial species that play key roles in visual turf quality of an established mixture turf.

## Data Availability Statement

The original contributions presented in the study are included in the article/Supplementary Material, further inquiries can be directed to the corresponding authors.

## Author Contributions

FX, GC, and YC conceived and designed the study. FX, GZ, QZ, XY, ZS, and KL performed the experiments. FX, XS, SS, LZ, RY, and WD analyzed the data. FX, GZ, QZ, and ZS wrote the first draft of the manuscript. KL, XS, RY, and WD prepared figures and tables. FX, SS, LZ, XY, GC, and YC revised the manuscript critically for important intellectual content. All authors approved the final manuscript to be published.

### Conflict of Interest

KL was employed by Beijing Oriental Garden Environment Co., Ltd.

The remaining authors declare that the research was conducted in the absence of any commercial or financial relationships that could be construed as a potential conflict of interest.
