# Supplementary material for: Beneficial Effects of Mixing Kentucky Bluegrass With Red Fescue via Plant-Soil Interactions in Black Soil of Northeast China
Source: Front Microbiol. 2020 Oct 28;11:556118. doi: 10.3389/fmicb.2020.556118 (PMC7656059; doi:10.3389/fmicb.2020.556118)
Supplement: Supplementary file 1 [file Table_1.docx]

Table. S1 Comprehensive evaluation value and size ranking of each sowing treatment

| Sowing models | PP | PC | PF | PCF |
| --- | --- | --- | --- | --- |
| Comprehensive value | 0.766 | 0.627 | 0.858 | 0.725 |
| Comprehensive sequence | 2 | 4 | 1 | 3 |
| Rank | II | II~III | I | II |
| Evaluation of lawn quality | Good | Medium | Fine | Good |

PP: 100% Kentucky bluegrass ‘Midnight’; PC: 50% Kentucky bluegrass ‘Midnight’ +50% Red fescue 'Barlineus'; PF: 50% Kentucky bluegrass' Midnight'+ 50% Red fescue ‘Frigg’; PCF: 50% Kentucky bluegrass ‘Midnight’+25% Red fescue ‘Barlineus’+ 25% Red fescue ‘Frigg’.
