# Supplementary material for: Beneficial Effects of Mixing Kentucky Bluegrass With Red Fescue via Plant-Soil Interactions in Black Soil of Northeast China
Source: Front Microbiol. 2020 Oct 28;11:556118. doi: 10.3389/fmicb.2020.556118 (PMC7656059; doi:10.3389/fmicb.2020.556118)
Supplement: Supplementary file 2 [file Table_2.docx]

Table S2 The percentages of rust (*Puccinia* spp.), powdery mildew (*Erysiphe graminis*) and brown spot (*Rhizoctonia solani*) at different growth stages in PF mixture and PP monoculture

| Month | Rusts (%) | | Powdery mildew (%) | | Brown spot (%) | | |
| --- | --- | --- | --- | --- | --- | --- | --- |
|  | PP | PF | PP | PF | PP | PF |  |
| July | 6 ± 0.22 | 1 ± 0.01 | - | - | 18 ±0. 23 | 6 ± 0.12 |  |
| August | 8 ± 0. 36 | 1 ± 0.04 | - | - | 10 ± 0.12 | 0.5 ± 0.01 |  |
| September | 30 ± 0.78 | 4 ± 0.06 | 13 ± 0.28 | 3 ± 0.01 | 8 ± 0.31 | - |  |
| October | 15 ± 0.21 | 1.6 ± 0.02 | 8 ± 0.19 | 1 ± 0.01 | 3 ± 0.01 | - |  |
