# Supplementary material for: Beneficial Effects of Mixing Kentucky Bluegrass With Red Fescue via Plant-Soil Interactions in Black Soil of Northeast China
Source: Front Microbiol. 2020 Oct 28;11:556118. doi: 10.3389/fmicb.2020.556118 (PMC7656059; doi:10.3389/fmicb.2020.556118)
Supplement: Supplementary file 3 [file Table_3.docx]

Table. S3 Quality sequence and mean length of fungi and bacteria from PF mixture and PP monoculture

|  | Sowing models | quality sequences | Mean_len |  | quality sequences | Mean_len |
| --- | --- | --- | --- | --- | --- | --- |
| Fungi | PF | 61544 | 238.32 | Bacteria | 62187 | 416.34 |
|  |  | 60585 | 251.55 |  | 71303 | 416.82 |
|  |  | 57578 | 243.5 |  | 52704 | 416.01 |
|  |  | 71946 | 241.14 |  | 53507 | 416.23 |
|  | PP | 42732 | 232.6 |  | 45502 | 413.9 |
|  |  | 44512 | 232.71 |  | 57217 | 414.22 |
|  |  | 47750 | 234.47 |  | 57631 | 414.38 |
|  |  | 45315 | 234.6 |  | 49726 | 414.3 |
|  | Mean | 53995 | 238 |  | 56222 | 415 |
