# Supplementary material for: Beneficial Effects of Mixing Kentucky Bluegrass With Red Fescue via Plant-Soil Interactions in Black Soil of Northeast China
Source: Front Microbiol. 2020 Oct 28;11:556118. doi: 10.3389/fmicb.2020.556118 (PMC7656059; doi:10.3389/fmicb.2020.556118)
Supplement: Supplementary file 4 [file Table_4.docx]

Table S4 Relationships between the soil properties and soil enzyme activities

|  | UE | CE | APE | IE | SOM | NH_4_^+^-N | AP | AK |
| --- | --- | --- | --- | --- | --- | --- | --- | --- |
| UE | 1 | 0.73 | 0.83 | 0.97* | 0.95* | -0.63 | 0.52 | 0.37 |
| CE | 0.73 | 1 | 0.99** | 0.69 | 0.86 | -0.35 | 0.73 | 0.34 |
| APE | 0.83 | 0.99** | 1 | 0.84 | 0.98* | -0.52 | 0.74 | 0.31 |
| IE | 0.97* | 0.69 | 0.84 | 1 | 0.96* | -0.74 | 0.67 | 0.23 |
| SOM | 0.95* | 0.86 | 0.98* | 0.96* | 1 | -0.71 | 0.68 | 0.27 |
| NH_4_^+^-N | -0.63 | -0.35 | -0.52 | -0.74 | -0.71 | 1 | -0.41 | 0.11 |
| AP | 0.52 | 0.73 | 0.74 | 0.67 | 0.68 | -0.41 | 1 | -0.32 |
| AK | 0.37 | 0.34 | 0.31 | 0.23 | 0.27 | 0.11 | -0.32 | 1 |

Note: *and**represent significant difference between the soil properties and soil enzyme activities (P < 0.05 and P < 0.01, respectively). Urease enzyme (UE), catalase enzyme (CE), alkaline phosphatase enzyme (APE), invertase enzyme (IE).
