# Supplementary figures and images for: Beneficial Effects of Mixing Kentucky Bluegrass With Red Fescue via Plant-Soil Interactions in Black Soil of Northeast China
Source: Front Microbiol. 2020 Oct 28;11:556118. doi: 10.3389/fmicb.2020.556118 (PMC7656059; doi:10.3389/fmicb.2020.556118)

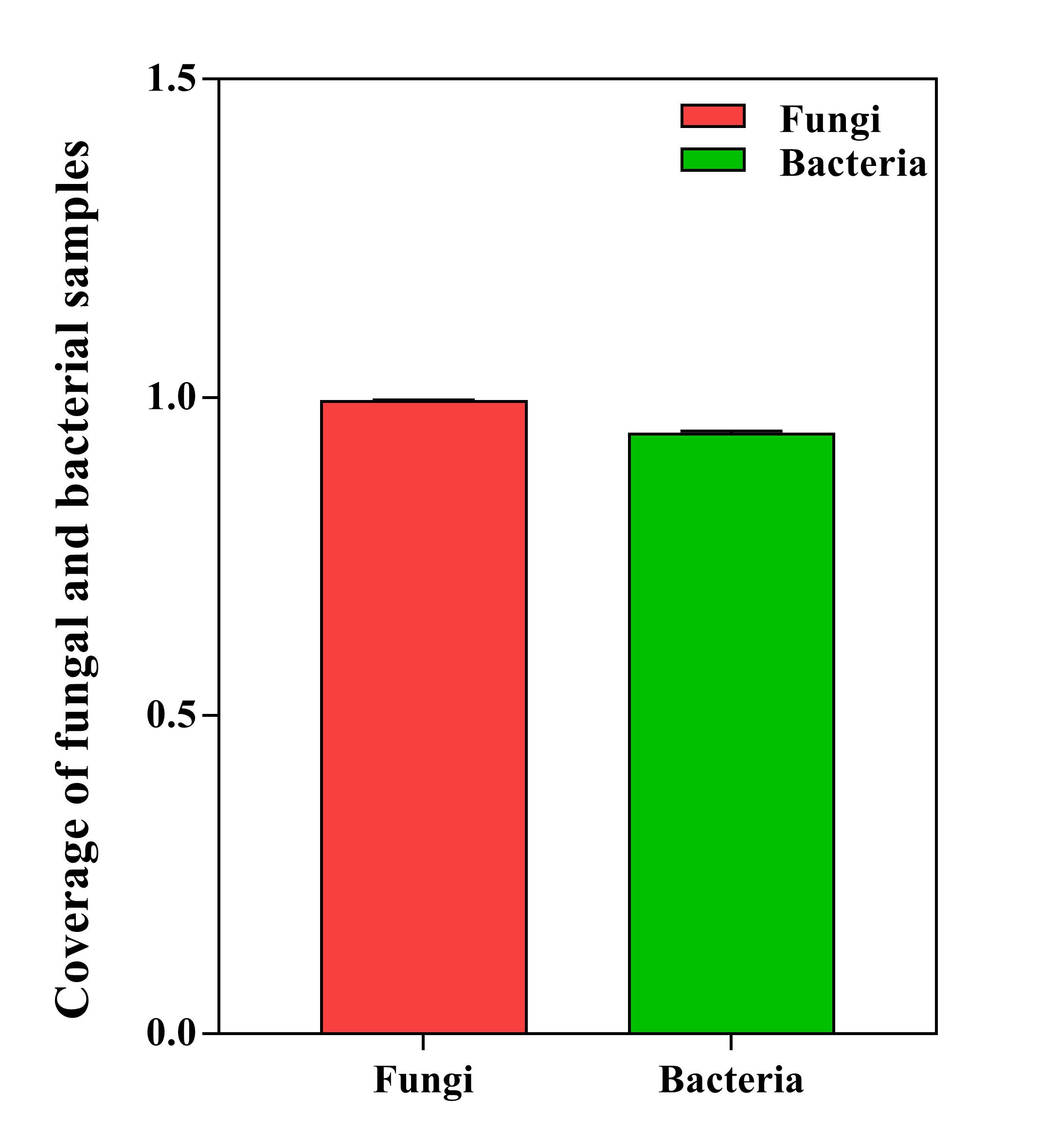

Supplement: Supplementary file 5 [file Image_1.jpg]

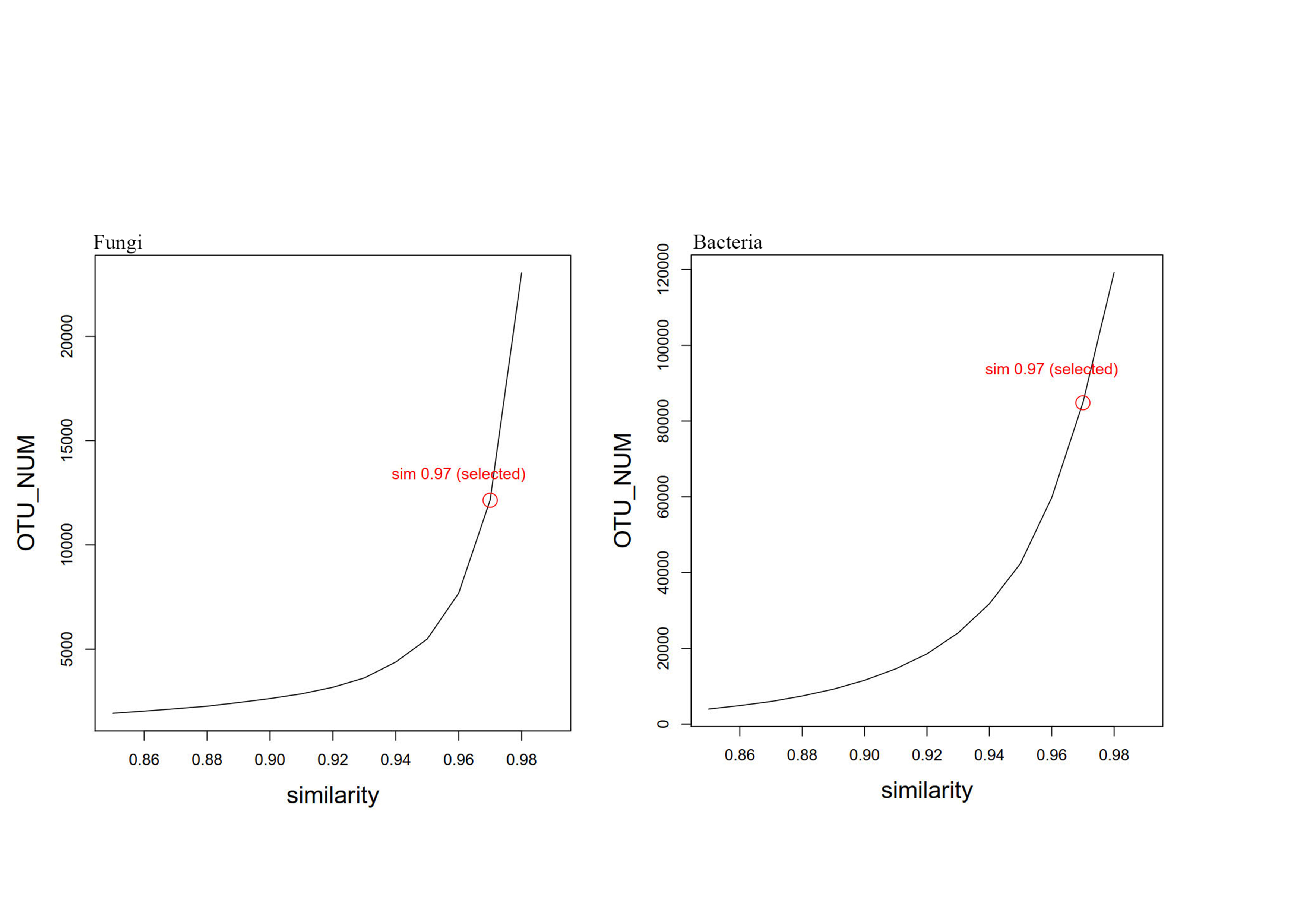

Supplement: Supplementary file 6 [file Image_2.jpg]

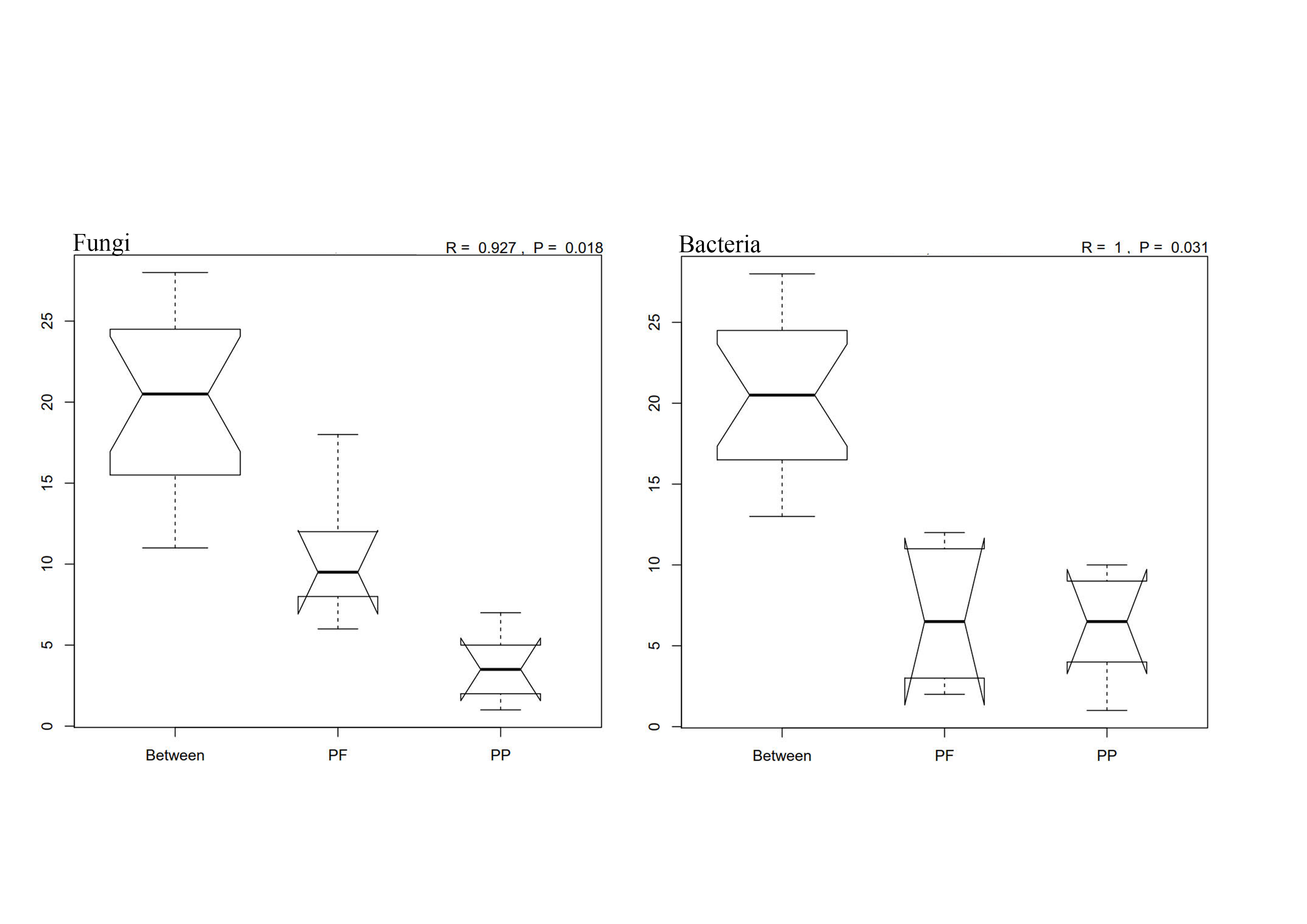

Supplement: Supplementary file 7 [file Image_3.jpg]

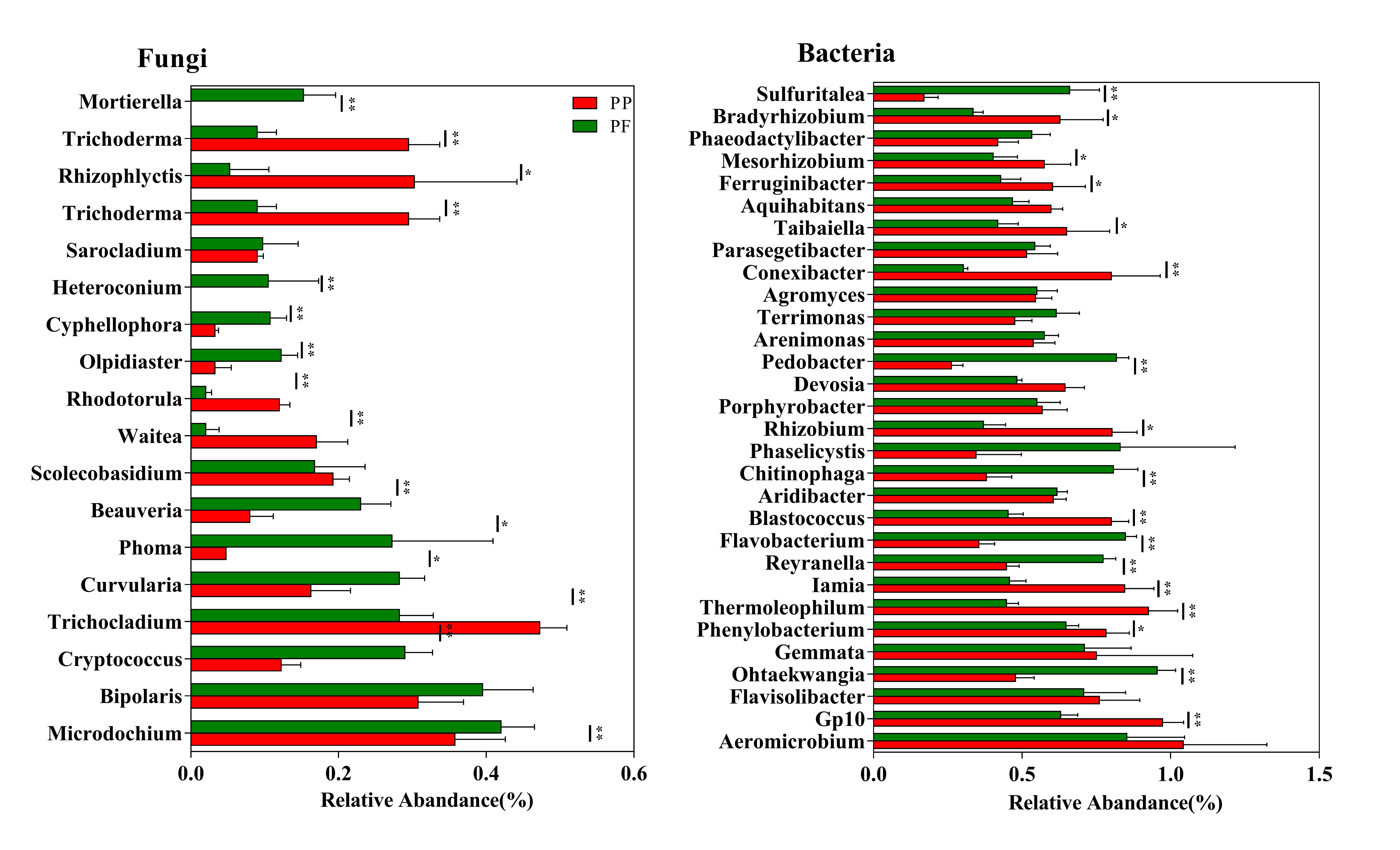

Supplement: Supplementary file 8 [file Image_4.jpg]

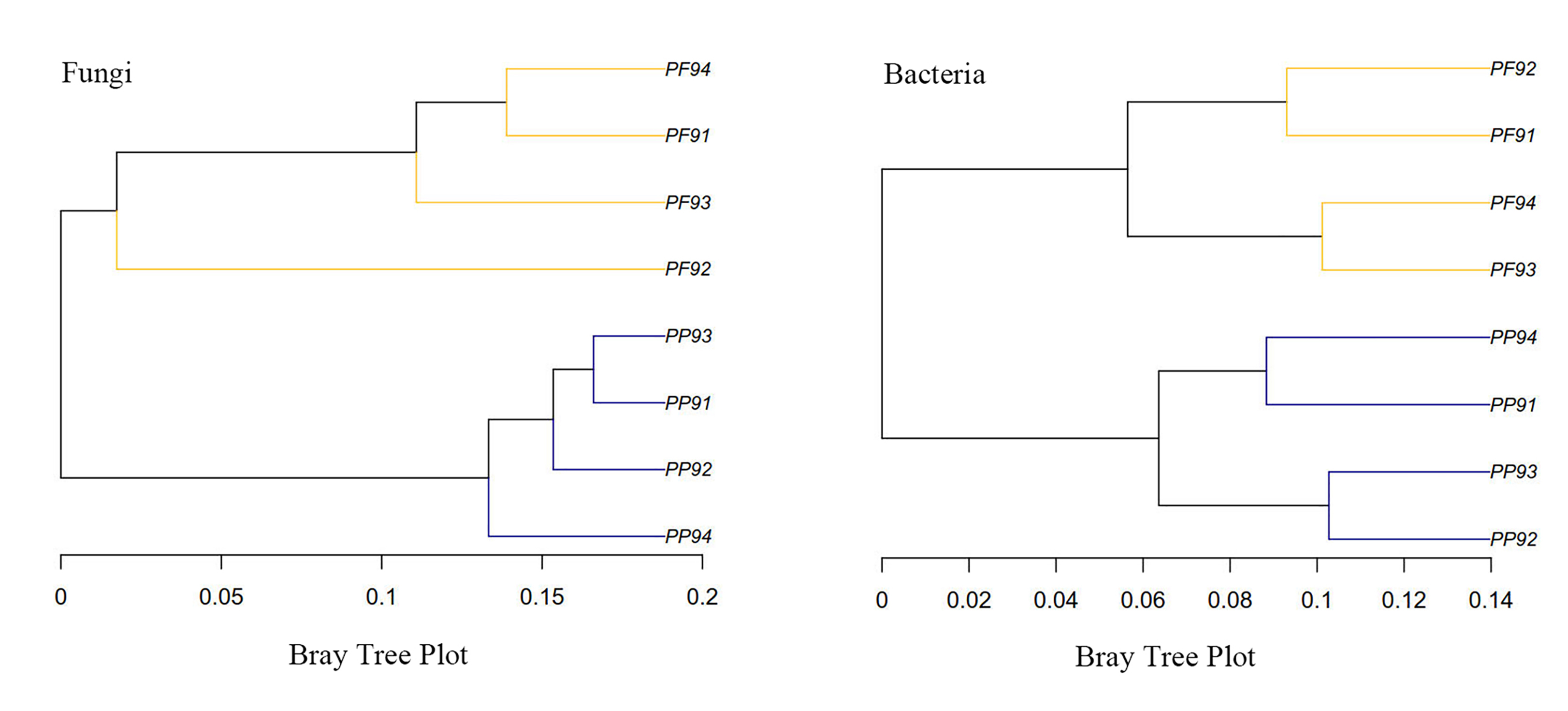

Supplement: Supplementary file 9 [file Image_5.jpg]
